# Selection Bias in Strong Coupling Experiments

**DOI:** 10.1021/acs.jpclett.3c03546

**Published:** 2024-02-15

**Authors:** Philip A. Thomas, William L. Barnes

**Affiliations:** Department of Physics and Astronomy, University of Exeter, Exeter EX4 4QL, United Kingdom

## Abstract

The strong coupling of light and molecules offers a potential new pathway to modify the properties of photonic modes and molecules. There are many reasons to be optimistic about the prospects of strong coupling; however, progress in this field is currently hindered by challenges in reproducibility, problems associated with differentiating between strong coupling and other effects, and the lack of a clear theoretical model to describe the reported effects. Concerning the question of differentiating between strong coupling and other possible mechanisms when examining experimental data, here, we show how cognitive bias can lead us to place undue emphasis on a given interpretation of unsystematic experimental data. We hope that this Viewpoint will, where appropriate, help readers to plan strong coupling experiments more carefully and evaluate the significance of the data obtained from them.

The strong coupling of light and molecules is a fascinating, rapidly growing area of research. It considers coupled light and molecular states where the coupling strength is high enough to replace the uncoupled light and molecular modes with hybrid light–matter states known as polaritons.^1^ Strong coupling therefore provides a potential pathway by which a variety of photonic and molecular properties might be modified.^2^

The prospects of strong coupling are clearly exciting, but the field faces challenges. For example, while strong coupling may well influence results obtained from transient absorption spectroscopy,^3^ Raman spectroscopy,^4^ nonlinear optics,^5^ and reverse intersystem crossing,^6^ subsequent work has identified additional nonpolaritonic effects in these experiments that complicate their analysis.^7−10^ The field of polaritonic chemistry (which aims to modify chemical processes with strong coupling) is at an interesting and perhaps pivotal stage. Many exciting results from experiments have been reported, but a clear and comprehensive theoretical understanding of these effects has yet to be developed.^11−13^ In addition, questions have arisen concerning reproducibility. Two early published attempts at reproducing polaritonic chemistry results were unsuccessful.^14,15^ Since then, two experiments have been successfully reproduced,^16−19^ although in one case it was found that the observed effects could be explained without invoking strong coupling^18,19^.

While we remain very optimistic about the prospects of strong coupling, we nevertheless agree with a recent review arguing that the field would benefit from a re-examination of the basics.^20^ Here, we draw attention to the confounding role cognitive bias can play in the design and interpretation of strong coupling experiments. If experiments are conducted in an unsystematic manner, cognitive bias can influence the design of an experiment, which might in turn influence our interpretation of the data so obtained.^21^ One example, and the one we focus on here, is selection bias: if we expect to see a change in a measured parameter (such as reaction rate constant) under conditions for strong coupling, we might be tempted to make most of our measurements under the conditions for strong coupling with just a handful of measurements under conditions where strong coupling is not present. When examining the results from this experiment, this selection bias can give the impression that something is happening around the condition for strong coupling simply because that is where most measurements have been made.

We illustrate this problem in Figure 1 using data from our own recent work.^19^ In this experiment, the rate of photoisomerization of a photochromic molecule inside a planar cavity was measured for a range of cavity thicknesses. The cavity thickness can be tuned to give strong coupling between the cavity’s photonic mode and the photochromic molecule product’s molecular resonance at 2.2 eV. Further details can be found in ref (19). In Figure 1a we plot a subset of data (a higher decay constant means more rapid photoisomerization), with a vertical dashed line indicating the cavity thickness for strong coupling with zero detuning between the first-order cavity and molecular modes (i.e., when the cavity mode energy perfectly matches the molecular mode energy). Based on this plot it is tempting to suggest that the photoisomerization decay constant is lower under conditions of strong coupling, but that impression is only given by the higher decay constants of the 122, 178, and 199 nm cavities. We do not know if these points are outliers or if the minimum truly falls at the cavity thickness for zero-detuning strong coupling. We are thus unable to say if photoisomerization is suppressed by strong coupling at around 160 nm or enhanced by another effect at around 120 and 180 nm. Crucially, we are unable to determine any trends that might confirm or rule out the role of strong coupling or other nonpolaritonic effects. While the data in this plot are *consistent* with the idea that strong coupling suppresses photoisomerization rates, they are insufficient to confidently *establish* a causal link between strong coupling and photoisomerization rates.

**Figure 1:**
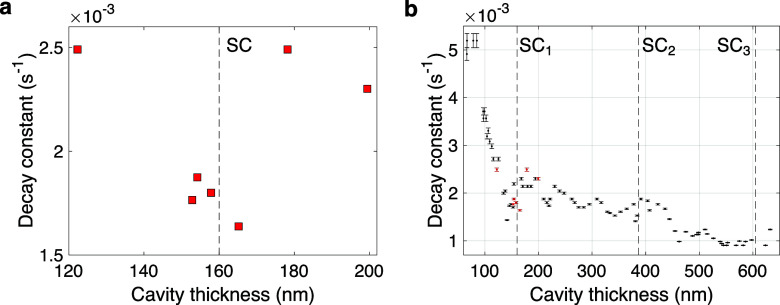
Results from a cavity-modified photochemistry experiment.^19^ The rate at which a photoisomerization process proceeded inside a planar cavity was measured for cavities with a range of thicknesses. Higher decay constant indicates more rapid photoisomerization. (a) Photoisomerization for a subset of measured cavities, around the cavity thickness under which strong coupling occurs (indicated by the vertical dashed line SC). This plot appears to show suppressed photoisomerization rates around the condition for strong coupling, but this is insufficient to establish a causal link between strong coupling and photoisomerization. (b) The full data set, taken for a wider range of cavity thicknesses that encompasses cavities that give strong coupling at three thicknesses (indicated by the vertical dashed lines SC_1,2,3_). There is a clear trend in photoisomerization as cavity thickness is varied, but it does not appear to correlate with the conditions for strong coupling.

Our fuller data set is plotted in Figure 1b: the points previously plotted in Figure 1a are in red, and the vertical dashed lines indicate the cavity thicknesses required for zero-detuning strong coupling to the first-, second-, and third-order cavity modes (labeled SC_1,2,3_ respectively). This data set is the result of systematic experimentation, with no bias in favor of any particular cavity thicknesses, allowing one to identify trends in photoisomerization as cavity thickness is varied. This plot confirms that there is indeed a minimum in decay constant close to SC_1_, but the lack of any comparable drop in decay constant around both SC_2_ and SC_3_ challenges the idea that it is caused by strong coupling. After further investigation, we found that this trend could be best explained by how efficiently the cavity absorbs ultraviolet radiation.^19^

We suggest that a systematic approach to experiments is essential for their results to inspire confidence. When scrutinizing experimental results in the literature, we encourage readers to ask themselves these questions: Perhaps these results are consistent with the authors’ conclusions, but would the claimed effect be obvious in the presented data without the authors’ commentary? Are the results systematic enough to rule out other explanations? If not, the authors’ instincts might be correct, but the experimental data are insufficient to justify their claims.
